# ‘Are you siding with a personality or the grant proposal?’: observations on how peer review panels function

**DOI:** 10.1186/s41073-017-0043-x

**Published:** 2017-12-04

**Authors:** John Coveney, Danielle L Herbert, Kathy Hill, Karen E Mow, Nicholas Graves, Adrian Barnett

**Affiliations:** 1 College of Nursing and Health Sciences, Flinders University, Adelaide, Australia; 2 School of Public Health, Social Work & Institute of Health and Biomedical Innovation, Queensland University of Technology, Brisbane, Australia; 3 School of Nursing and Midwifery, University of South Australia, Adelaide, Australia; 4 University of Canberra, Canberra, Australia

**Keywords:** Peer review process, Black box, Research funding, Grant proposal, Grant review panels

## Abstract

**Background:**

In Australia, the peer review process for competitive funding is usually conducted by a peer review group in conjunction with prior assessment from external assessors. This process is quite mysterious to those outside it. The purpose of this research was to throw light on grant review panels (sometimes called the ‘black box’) through an examination of the impact of panel procedures, panel composition and panel dynamics on the decision-making in the grant review process. A further purpose was to compare experience of a simplified review process with more conventional processes used in assessing grant proposals in Australia.

**Methods:**

This project was one aspect of a larger study into the costs and benefits of a simplified peer review process. The Queensland University of Technology (QUT)-simplified process was compared with the National Health and Medical Research Council’s (NHMRC) more complex process. Grant review panellists involved in both processes were interviewed about their experience of the decision-making process that assesses the excellence of an application. All interviews were recorded and transcribed. Each transcription was de-identified and returned to the respondent for review. Final transcripts were read repeatedly and coded, and similar codes were amalgamated into categories that were used to build themes. Final themes were shared with the research team for feedback.

**Results:**

Two major themes arose from the research: (1) assessing grant proposals and (2) factors influencing the fairness, integrity and objectivity of review. Issues such as the quality of writing in a grant proposal, comparison of the two review methods, the purpose and use of the rebuttal, assessing the financial value of funded projects, the importance of the experience of the panel membership and the role of track record and the impact of group dynamics on the review process were all discussed. The research also examined the influence of research culture on decision-making in grant review panels. One of the aims of this study was to compare a simplified review process with more conventional processes. Generally, participants were supportive of the simplified process.

**Conclusions:**

Transparency in the grant review process will result in better appreciation of the outcome. Despite the provision of clear guidelines for peer review, reviewing processes are likely to be subjective to the extent that different reviewers apply different rules. The peer review process will come under more scrutiny as funding for research becomes even more competitive. There is justification for further research on the process, especially of a kind that taps more deeply into the ‘black box’ of peer review.

## Background

Health and medical researchers across the world submit their ideas to peer review to gain funding. Competition for research funding is intense, as success rates in most schemes are low and careers are on the line. The opinions of peer reviewers can mean the difference between success and failure in securing funding and publications [1]. The peer review process in Australia mostly uses face-to-face meetings of reviewers combined with prior assessment from external assessors. It takes time and money to assemble the people and information required for peer review, including applicants’ time (including their institutional administrative support), peer reviewers’ time and administration at the funding agency. Commentary on and criticism of the peer review process have been raised for many years [2–4]. Examples include concerns about usefulness in predicting overall research output and high costs [5], overall workability [6] and difficulties with conflicts of interest [7]. Others, however, document that the peer review processes can identify the most promising proposals in terms of research productivity [8].

In Australia, the two most common sources of national competitive funding are the Australian Research Council (ARC) and National Health and Medical Research Council (NHMRC), and both use peer review panels to assess applications. Anecdotal evidence abounds discussing the dynamics of peer review panels and the membership of panels. Of particular interest are the factors that are considered crucial to an application’s success. Indeed, one of the advantages of being a panel member is gaining first-hand knowledge of the so-called black box [9], that is, the decision-making process that assesses the excellence of an application. For those who do not have direct experience of panel membership, gaining insights into the ‘black box’ can be difficult, even mystifying. While the criteria for judging the quality of applications are freely available to applicants (e.g. significance, track record), there is surprisingly little information on the dynamics of panel members in the peer review process and the factors that are taken into account in supporting or sinking an application. Mow’s research into the ‘black box’ of peer review almost stands alone as an examination of the process from the perspective of peer reviewers. The research reported here extends Mow’s findings by shedding further light on the grant review panellists and their experience of the ‘black box’.

### The larger study

This project reported here was nested within a larger study examining the costs and benefits of a simplified peer review process. A full description of the larger study is available at [5], but in brief, Australian researchers who had submitted a NHMRC proposal in 2013 were invited to provide the proposal to our team. We received 145 proposals. We narrowed these down to the key fields of Basic Science and Public Health which each had 36 proposals. Basic Sciences and Public Health were chosen because an earlier NHMRC study showed that each correlated differently on the basis of bibiometric measures, with Basic Science having high correlation and Public Health having low correlation. For this project, Basic Sciences included biochemistry, immunology and cell biology; Public Health included epidemiology, health promotion and disease prevention and health economics.

#### Expert review panels

Two seven-person expert panels reviewed a sample of the proposals in separate 1.5 day face-to-face meetings. In general, the panellists were the lead investigator for each proposal. The primary aim of the panels was to test a Queensland University of Technology (QUT)-simplified process by reviewing shortened proposals, including a nine-page research plan and two-page track record for each investigator. Each panel member was a spokesperson for five or six proposals, and they gave an opening summary of the strengths and weaknesses of the proposal based on a review they had prepared prior to the meeting.

### This study

The overall aim of the research reported here was to explore the experiences of panel members while serving on grant review panels and to reflect on this experience in light of the QUT-simplified process. That is to say, we were interested in views about the impact of the panel procedures, composition and dynamics on the panel’s decision-making in the QUT-simplified process, but we also wanted to gather participants’ views on other panels, for example, NHMRC grant review panels (GRPs).

The objectives of the study reported here were:

1. To explore experiences of members while serving on grant review panels, in particular what factors were thought to determine ranking of applications—essentially what was thought to constitute a high-quality proposal—and how to deal with conflict of interest.
2. To compare experiences of the QUT-simplified approach with other more standard panel processes. In particular, the extent to which the QUT-simplified process provides a more efficient and less demanding assessment procedure while continuing to be robust and discriminating.
3. To observe any differences between the two panels that may reflect different research cultures and the relevance for grant assessment and appraisal. In particular, the extent to which the underlying expectations of each group may be revealed by contrast and comparison.

## Methods

The expert panellists generally had experience of varying degrees in (a) winning funding from NHMRC, (b) serving on NHMRC grant review panels and (c) being an external reviewer for NHMRC. Of the 16 panellists (14 panel members and 2 observers), 12 were at Professor (Academic Level E) or Associate Professor (Academic Level D).

Each panel member was invited to be interviewed about their views on in the QUT-simplified process and their experiences as members of other grant assessment panels. Some respondents chose to be interviewed immediately after the QUT panel completed its assessment. Other panel members were interviewed at a later date, but within 4 weeks of the QUT panel meeting. No panel member refused to be interviewed.

All interviews were conducted by two of the authors (JC and KM) and were recorded and transcribed (DH). Each transcription was de-identified and returned to the respondent for review (DH), the so-called member’s check [10]. All final transcripts were read repeatedly and coded [11]. Similar codes were amalgamated into categories [12], and categories were used to build themes. Themes emerging early representing broader categories were identified and discussed by two authors (JC and KM) for clarification and relevance. Final themes were shared with the research team for feedback.

## Results

Here we report on two major themes (and subthemes) that emerged from the interviews with the QUT panellists. The themes and subthemes are:

1. Assessing grant proposals

- What makes a good grant proposal?
- Assessing grants: QUT method versus NHMRC
- Judging value for money: QUT method versus NHMRC
- ‘Major players’ large share reduces capacity building

2. Factors affecting the fairness, integrity and objectivity of the review

- The importance of experience of panel membership
- Ensuring a fair review
- The role of track record
- The purpose and use of the rebuttal
- The impact of the group dynamics on grant review processes

We also report on what were seen to be major differences between the two panels, Basic Sciences (BS) and Public Health (PH), which we believe demonstrates cultural, or epistemological, differences between the two groups. The quotes are referenced for each panel member as either BS or PH according to the respective peer review panel.

### Assessing grant proposals

#### What makes a good grant proposal?

##### Clear writing

There was uniform agreement across both grant review panels that the success of a grant depended highly on the quality of the writing. The ideal was a clear and simple proposal that could be understood by someone not necessarily from that particular field;

1BS 6 ‘I haven’t yet seen a well written grant application that didn’t improve its score by just having been readable, […]^2^ as you’re reading it, it’s like a whodunit almost. it’s that wonderful balance of appropriate diagrams with clear text which will have you in their pocket basically’.

##### Good science and translation

Both panels were also able to express a common expectation that the grants had to represent good science regardless of the discipline. There were, however, key differences in the panels’ opinions as to what constituted good science. The Basic Science group placed greater emphasis on ‘innovation’ and ‘novel’ proposals, while the Public Health panel allowed more flexibility and was more ready to accept a generic type of science that would be making a contribution to population health overall with some degree of translation;

BS 2 ‘I think that the proposals that rank the highest… they’re stretching the boundaries of where we are in the research at the time’.

PH 6 ‘All grant writing is selling and I'm not interested in how clever you are or how intellectual you can be; I just want good, solid, doable research which is going to make a difference’.

For the Basic Sciences group, it was not necessary for the research to be immediately translated from the laboratory to the bed side; it was adequate if the panel could see the value of the proposal in light of its contribution to the incremental approach of Basic Science research, with each small part contributing to a larger whole of global research.

#### Gold (should fund), silver (could fund), bronze (should not fund) versus NHMRC 1 to 7: which is best?

One unique aspect of the assessment of grants within the QUT-simplified process was the introduction of a parallel scoring system. In the NHMRC, process grants are scored from 1 to 7. The QUT-simplified process entailed only three categories—gold (should fund), silver (could fund) or bronze (should not fund). Both panels, with almost complete consensus, believed that the simplified system vastly improved the process;

BS 4 ‘the [NHMRC] seven point rating does obfuscate one’s thinking… the human species as a whole – does think in clear categories: fund, maybe fund, not fund…. it was very helpful to use that score, you know, one, two, three; gold, silver, bronze’.

In addition, the overly detailed 1 to 7 NHMRC grading was often criticised;

BS 8 ‘Oh, I think both systems have good things and bad things. So 1 to 7 is stupid because of human nature. Unless you hate someone, why would you give out a 1?’

However, there was also some suggestion that, in a similar way to the NHMRC, this three-tiered ranking process left a large number of grants in the ‘middle ground’;

PH 4 ‘Most of the grants that are brilliant, the fallout is brilliant and most of the crap falls out as crap and then, as we discussed, over time you’ve got this grey area where it’s a bit of a lottery’.

A number of participants suggested that to address this, some levelling of silver into two categories was warranted;

PH 3 ‘I think that it [QUT system] is great, that’s a much better system. I would like to see a 2A and a 2B, like, you know, polished silver and tarnished silver or something along those lines’.

However, there was a high degree of acceptance of a simplified system with only one dissenting opinion, reported;

BS 6 ‘Well, it comes down to a head counting exercise with how many – what proportion of the people are giving it a gold score as opposed to a silver or a bronze? It’s lost a resolution’.

#### Budget and value for money (QUT v NHMRC)

Another key omission in the formulation of the applications for the QUT-simplified process was project budgets. In addition to this, the concept of ‘value for money’ was introduced into the interviews for the panel members to consider. There was significant divergence of opinion with most of the Basic Science panel feeling that if the science was worth funding, then it should be funded regardless of the budget and that value for money should not be given any consideration;

BS 2 ‘Yeah, I don’t really take the funding into account; it’s a separate entity. …if we decide that we’re going to fund high quality science we should fund the science and not cut the budgets so that they can only do half the experiments’.

BS 8 ‘So if we start putting a value on top of this kind of judgment, how do you judge your research, you know? How much is it worth? It’s stupid. It’s worth nothing and millions of dollars’.

In the instances when the Basic Science group considered budget, it was only in terms of whether or not the science was valid and there was a sense of responsibility to use the available funding wisely;

BS 6 ‘[I] think you have to be – unless the potential outcomes were commensurate with the expenditure I think it doesn’t make common sense and it wouldn’t be responsible to be advocating public monies to be spent – it’s not as if there’s a surplus of money so why would we pour money into an idea that can’t justify that then because there’s no shortage of alternative destinations for that money’.

Within the Public Health panel, although there was clear acknowledgement that the science is judged first, there was majority reasoning that the budget could play a role in achieving funding; thus, its absence was felt to hinder their decision-making. This panel also largely felt that in health research, ‘value for money’ is an important consideration;

PH 4 ‘And everybody kept asking […] for the budget, you know “it would really help me to see the budget. Is this a million dollars, in which case it’s bullshit, or is it $10k in which case we should definitely support it as a pilot?” …I know that the budget isn’t supposed to influence your decision making but it does, even if subliminally’.

One aspect of this particular theme in which both groups reached consensus, however, was the difficulty that can be experienced in achieving grant success for a study with a very large budget;

PH 3 ‘I understand that at some point it’s important to consider that but I wouldn’t like to be in the situation where we say “okay, so this grant’s pretty good but it’s a million dollars. This grant’s not so good and it’s only $200,000; let’s fund that [$200,000] one”. I don’t think that that’s appropriate, or if we think that this is important we should be funding it, but I think in general people do feel quite nervous about really big budgets.’

There seemed to be agreement in the Basic Sciences panel that most grant writers manipulated the budget to some extent in anticipation of the NHMRC either viewing a smaller budget favourably or conversely the applicant increasing the budget in anticipation of an NHMRC reduction;

BS 4 ‘Downgrading your budget is a common fault. Upgrading your budget is also a common fault and that’s done by the more experienced players in the field who’ve long since come to the realisation well, you know, NHMRC will cut you back anyway almost certainly so you inflate your budget a bit’.

BS 5 ‘You know, you put something in there that’s not going to break the project that they can easily cut off. Everyone talks about that unofficially, yes’.

#### ‘Major players’ large share reduces capacity building

There was some agreement, most notably amongst the Basic Sciences panel, that a larger organisation with a strong track record and with ‘major players’ on their teams attracted the lion’s share of the funding which in turn, they acknowledged, disadvantaged early career researchers;

BS 3 ‘They have an enormous amount of awe of these people, right, and so even in the normal GRP they get a really easy run because everybody thinks they’re there, they must be fantastic and I suppose what I think is if that’s the case why have a GRP? Just give them the money’*.*

PH 4 ‘As I say, it’s basically just going to be the spinners that are going to do better and I’d rather make that decision myself … there is negativity about the fact that you cannot - in a system structured to support that type of activity, you cannot then support brilliant, innovative young minds who aren’t necessarily affiliated with the right group, haven’t got the ten publications, don’t know enough people who know people on the review panel for their fantastic stuff to get up’.

It was also discussed that being a panel member could confer a certain advantage in terms of an individual’s grant success and a number of participants spoke very highly of being involved in a panel for mentoring, career growth and the ability to produce a higher quality grant.

### Factors influencing the fairness, integrity and objectivity of review

#### Ensuring a fair review

##### Expert ‘out of the room’

One of the most serious and repeated criticisms from panellists in this study was of the NHMRC process where the conflict of interest regulations almost always ensure that the vital expert opinion, required to ensure an adequate and fair peer review, has left the room;

BS 7 ‘we have had anecdotes where every single expert in the country has to leave the room, even for minor things’.

A number of respondents felt that if you were working in a highly specialised field and ‘your’ expert left the room the inability of the panel to then extrapolate the key points in the grant was something that almost inevitably resulted in the grant faring badly;

PH 8 ‘The people who excused themselves were the people with the discipline background and to exclude them from the review of these particular papers really didn’t do the grants any service at all because those people who knew that area were not there’.

##### External reviews

In contrast to the criticism of this overly rigorous process, the two panels both subsequently questioned the continued usefulness of the NHMRC soliciting an external review, its actual legitimacy and the reviews’ potential impact on grant success. It was thought that as procuring the reviews is no longer the responsibility of the spokesperson to complement their own assessment, this has significantly reduced their usefulness;

PH 4 ‘I think it’s the primary spokesperson’s synthesis that really carries most weight and the [external] reviewer’s comments are used only rarely to say, you know “how can you say that when the Nobel Prize winner said it was brilliant?” or vice versa. So they are useful but the impost on the reviewers to write them and the second spokesperson to interpret them and report back on them while everybody’s going to sleep at the table, particularly when some people do it line by line, it is not worth the effort’.

There is also some suspicion that an ‘expert’ review can be reasonably hard to obtain and the quality that is expected in these circumstances cannot always be relied on;

BS 4 ‘I’ve never been convinced in recent years about external reviews, largely because I know NHMRC has got a lot of difficulty getting people to write external review’.

PH 6 ‘The only experience I have got of external reviews is in the real thing and they are - if external reviews correlate with the primary spokesperson’s opinion, then they are normally included; but if they are against the primary spokesperson’s opinion, they are normally discarded’.

Both the conflict of interest regulation and the external reviews are a process designed to ensure that the grant receives a fair review. However, across both groups, there was a subtle but consistent suggestion that one person, with a carefully crafted sentence, could decide the fate of a grant and that the conflict of interest and external review designed to avoid this occurring often fails to do so. The related concepts of ‘sinking a grant’, ‘gaming the score’ and conversations ‘off the record’ will be further explored as subthemes later in this section.

##### Vital role of the spokesperson as an advocate for a grant

When discussing the role of a spokesperson, the respondents articulated that this is a responsibility they take very seriously. The QUT process mirrored the NHMRC process in the allocation of grants to each panel member, including the chair, for which the member was then expected to present to the group a synopsis of the key points in the grant including aspects that could be improved, or aspects that were considered exceptionally good;

BS 2 ‘if you’re the primary spokesperson and you don’t know the answer to a question it’s kind of your fault. You don’t have to know the answer to every question but you have to be on top of what’s contained in the grant’.

Across both groups, despite the notion that the grant review process would be equitable, was a strong sense of being an ‘advocate’ for a grant which highlights the importance of allocating the correct grant to the most appropriate spokesperson;

BS 5 ‘I guess those few grants that I really liked I did defend and push quite strongly. Yes, I did feel like an advocate’.

BS 8 ‘So that’s a problem that everyone has, I think, that when we don’t know what to do with it, we just put them in the big grey bag of the four or five-points grants; and then everybody rallies behind the spokesperson and that’s when you can push or kill a grant’.

The panel members spoke of sometimes undertaking extra research in their role of spokesperson to understand the grant more fully and to ensure that they gave each one a fair representation. Without doubt, both panels as a whole were guided by the spokesperson’s report into making a decision regarding the grant, deferring to them as the person that held the most information about the likely success of the proposal;

BS 3 ‘In many ways it’s a bit of a myth that of the six or seven reviewers around the table you get six or seven independent scores. You don’t because your scores will be influenced, you will bow to superior knowledge for the spokesperson who really knows what they’re talking about on a given grant’.

This process, as a result, makes the role of the spokesperson absolutely vital. There were at times, however, suggestions that if, for various reasons, the spokesperson did not take the role of the grant advocate, it then had little chance of a successful outcome;

BS 1 ‘The downside is if the primary spokesperson is tepid the grant’s got almost no chance of succeeding, right, because you’re looking for reasons to sink the grant and straight 5s [scores] is just a death knell. The grant’s dead, dead in the water’.

PH 6 ‘Well, it’s clearly critical. I felt very much it was about setting the expectations about this grant and if the spokesperson didn't like it, it is highly likely that everyone else would feel the most permission to go in hard and pick it to pieces’.

On some occasions, the spokesperson did highlight their lack of expertise on grants allocated to them in the QUT-simplified process, but most agreed this was due to the nature of the QUT configuration and was unlikely to occur on a NHMRC panel given the vital role that the spokesperson plays in the presentation of the grant;

PH 1 ‘I mean quite frankly I don’t think I was an expert in any of the six proposals [QUT simplified process] to which I was a spokesperson’.

BS 3 ‘That extrapolates into quite a thorny issue inasmuch as that if you haven’t got in-depth expertise in a particular proposal for which you are primary spokesperson’.

There was consensus in the groups that the NHMRC process is supposed to be regulated to ensure that the panel configuration will have the expertise to counter the effects of a spokesperson with limited expertise, which is then supported with a secondary spokesperson. This theme is explored next.

##### Experienced and appropriate panel

A key point made during this research is that the panel must be selected to ensure a broad range of experiences and also contain significant expertise. Some discussions of the NHMRC process within the Basic Sciences group suggested that the grant applicants at times felt the panel composition could have been improved in terms of level of expertise. This panel subsequently discussed at length the highly selective nature of the grants that would be reviewed during the NHMRC process—in which there is a immunology panel, a biochemistry panel etc.—and the rigour in which the panel members needed to be drawn together to ensure the grants received a fair hearing;

BS 2 ‘Yeah I think if panels are constructed well – and I can’t remember, we might have had three people speaking who had read the grants in depth so that means that three people really put their time and effort into that grant and if you’ve got the right breadth around the table then really good discussions happen and when that happens then the grants do really well’.

If at any point during the QUT process a member of the Basic Sciences panel thought that they lacked the experience to appropriately judge the grant, they said so emphatically and deferred to the judgement of the spokesperson. Most also reflected on taking this action during a NHMRC process;

BS 2 ‘I have been on panels where there was a grant that I said I wouldn’t provide a score for because I would just be making something up that was consistent with what the lead person’s score was. And while I trust that person I’m not just trying to balance out the scoring. I didn’t know the area at all. Even if I’d read for a week on it I still wouldn’t have known too much about it and so there was no reasonable way in which I could provide a score that would be meaningful’.

In contrast, the Public Health panel discussed a different approach to reviewing grants outside of their expertise during this process. They spoke of the panel being capable of reviewing a wider range of grants, including those that they would consider outside of their field. This difference perhaps is driven by the very different research paradigms with the very exact nature of Basic Science, and the more ‘broad-church’ approach seen in Public Health research.

PH 8 ‘Oh, hell, yes. Some were just so hard. Some of them, I didn't understand. I simply did not understand what they were looking at. And that was something I did learn from the other panel members. They were able to go directly to the science. So they were able to go directly to “let’s have a look at exactly what they want to do. Forget about all the background stuff for the moment. Let’s just look at the methods and look at what they want to do and how they want do it and what their outcomes are going to be.” So they were really clever’.

##### ‘Sinking a grant’

The potential for a single panel member to effectively influence the group into negatively viewing a grant was discussed within both panels. This occurred noticeably more so in the Basic Sciences panel, and although there is acknowledgement that the conflict of interest regulation is designed to avoid this occurring, the respondents clearly feel that it is not always successful in doing so;

BS 2 ‘I think you can load words and I think some people [in the QUT panel] did load their expression or the words that they used that really reflected that they have no enthusiasm whatsoever for the grant’.

BS 4 ‘the discussion will go on and on and up and down and sideways and it just takes one little whiff of a hint that there’s something wrong with the grant, not in a major way, just something – and your score goes down’.

BS 6 ‘There are people who seem to have a vindictive streak in them to try and cut other people in the field down and they don’t limit their vindictiveness necessarily to the weaklings, they go for anyone, you know’.

##### ‘Gaming the score’

Both panels discussed the manipulation of scores in order to counteract another panel member’s score or to influence the outcome of a particular grant. This was discussed on occasion in a fairly neutral and benign way in the Public Health panel and appeared to be in the context of ensuring a fairer outcome for a grant. But from within the Basic Sciences panel, the concept was highly repetitive, seen in the majority of the interviews often appeared to have a more serious intent;

BS 1 ‘The other way to do is you game the scoring system and that’s a really interesting process that gets used a lot. So that’s a more passive/aggressive way. You know, I’ve seen chairs gratuitously try to game the scoring system to get outcomes, like saying ‘well, let’s’ – you know, briefing their panel ‘we all know there’s going to be 15 per cent of grants we want to get up. To give our grants the best possible chance when they get ranked next to other people’s grants let’s give all of those a seven and anything we don’t want to fund a three and have a bimodal distribution’ so the scores went like that; nothing in the middle’.

BS 3 ‘it’ll be like “oh, wow, this is so great. We can go in there and sway everyone” and you think they won’t? They will. They absolutely will’.

PH 3 ‘I suspect in some of the other panels there’s lots of game playing and strategic assessing or strategic grading to get a lot of people up … so lots of them will be funded’.

##### ‘Off the record’

Another issue that arose during the interviews regarding the stringent management of conflict of interest is that it again at times fails to be effective and simply moves the conversation out of the panel discussion held in public into another area which then creates a conversation ‘off the record’;

PH 4 ‘I think everybody [at QUT panel], because they were empowered and trusted, were probably more objective than they would be at the real NHMRC panel where they’d be telling people in the coffee room ‘this guy’s a real bastard but, you know, I have to leave the room so make sure you sink it’ sort of thing’.

PH 1 ‘Well, it’s a matter of meeting procedure and often, as indeed at any scientific forum, the most influential conversations occur around the water cooler or coffee urn rather than in the sort of formal structure of the discussion’.

#### The role of track record

The role of track record is considered by these panels to be a very high priority when determining a successful grant from one that is not. The QUT process involved the provision of the two-page track record as prepared for the NHMRC panel (the NHMRC process includes far more details on papers and funding) and this created some mixed feelings;

PH 8 ‘Track record was very important; very important to the people who had been on the panels in the past, who had been on the real NHMRC panels’.

BS 8 ‘It is as good as admitting, you know. We are judging ego. It’s the most popular kid in high school that would get the best track record; it is polluting information. So I really appreciated that we did not have extra information on the candidates’.

##### Reputation in the field

Reputation in the field is an important factor that is considered in the review of the grant. There is general agreement that most people on the grant review prefer to know the candidates, or know of them, and know what they have achieved or where they ‘sit’ in their field. When this is not the case, and this is more often seen in the Basic Sciences panel, a person-centred judgement is still made which is then based on the institution that they are with and this process overall largely disadvantages early career researchers;

BS 1 ‘On this panel people wouldn’t be familiar where the applicants would lie in their field so that was the main hurdle that I saw for – there was one grant that I had that – I had no idea where that person fitted in their field, whether at the top, middle or the bottom’*.*

PH 3 ‘I think, based beyond the track record that you read in the grant, so you know like you’ve worked somewhere, you know those people or someone you know knows those people or you’ve just come across their name in lots of situations so you know that they’re very good’… ‘I think it can be really hard for early career researchers too and I know that, you know, they have the people support but it’s really – it’s even more competitive than project grants’*.*

BS 4 ‘Everybody knows who the worthy people really are… You all know each other and you all know who the bright ones are’.

A common point also made is that the addition of co-investigators or associate investigators with easily recognisable names is a smart strategy to attract the panel’s attention and increase the potential for the grant’s success;

PH 8 ‘They were big names and huge track records and their relevance for the project was really questionable. So whether the investigators thought that having those extra people on would give them more bang for their buck, I don’t know, but it was just so obvious that those people really weren’t going to play any role in the conduct of the research’.

##### Supervision of PhD candidates—capacity building

There was strong disagreement between the two panels on the value of a grant candidates’ supervision of research higher degree students. The vast majority of those in Basic Sciences considered this information irrelevant to the consideration of the grant; however, the Public Health panel members viewed this role as positive and considered it an essential part of a researcher obligation to contribute to supporting future researchers and capacity building;

BS 3 ‘For a project grant it’s completely irrelevant. It’s just a way of buffing their track record’.

PH 1 ‘I think that’s terribly important in the people scheme. You know, we want to support fellows, research fellows, senior research fellows who are training the next generation of researchers. Possibly a little bit less important in project grants but, you know, as a researcher and somebody who’s committed to building capacity it’s something that I like to see. If it’s a tossup on track record I will tend to favour the people who have made that investment in training the next generation of researchers’.

#### How useful is the rebuttal?

The formulation of the QUT panels omitted the opportunity for the applications to be externally reviewed and the related opportunity for rebuttal. This omission was explored in the interviews and there was a clear consensus in the Basic Science panel that the opportunity for rebuttal is useful in terms of refining your grant and trying to gain the confidence of the spokesperson;

BS 6 ‘it’s crucial that the applicant has the opportunity to rebut absolute nonsense’.

However, the general tone of both groups, and in particular the Public Health panel, suggested that most respondents believe that rebuttal rarely influences the spokesperson or the panel into a revision of their assessment and is perhaps more useful for the next round of submissions;

BS 5 ‘Would it [rebuttal] have changed any grant outcomes? I hear ‘no’ and I’ve just done my rebuttal [for the NHMRC process] and I don’t think it’s going to make any difference after spending a week on it. I mean it actually is a really good process to do yourself because you’re addressing what people might ask about your work but – and it’ll make your next grant submission better - but change of grant from not funded to funded, I don’t think it does’.

PH 1 ‘For the vast majority of the grants I don’t think it would have had any influence’.

#### The impact of the group dynamic on grant review

##### Role of the chair

Both the Basic Sciences and the Public Health panel members agree with a number of key principles regarding the role of the chairperson. Unanimously, respondents felt that a strong chair was needed to ensure that the group kept on task and dealt with the proposals fairly;

PH 4 ‘I think it’s a good chair who will make sure that the opportunity to say things is equal, particularly where you’ve got a difference in experience, gender, academic status and all of those things, you know better than I do, will influence who says what first. I had good chairs and I can remember them facilitating the process and improving the fairness of the scoring by quietening down openly vocal panel members and, again it’s around the table technique, and say “okay, thank you, we’ve heard from you now let’s hear from two more people before we go to scoring” and those sorts of techniques’.

Most agreed that the chair provided their expert opinion in exactly the same way as any other panel member if the grant came from within their area of expertise, which would, of course, influence the outcome of the grant but no more so than if any other expert were to present a highly valid judgement;

PH 3 ‘I think [pronoun]^3^ had an influence which was good in terms of [pronoun] was the person with content expertise’.

There were no respondents that felt the chair unduly influenced the outcomes of any grants, although there were some suggestions that this had occurred in the past prior to the more recent adaptation of a ‘technical chair’ in some cases rather than an expert from the field;

BS 6 ‘The first one I was on I don’t remember anyone being admonished but that didn’t mean that I didn’t think the system wasn’t – in other words I don’t think the chairperson was doing his job and that there were people who were very adversarial against projects that they didn’t like and conversely very advocating of ones they liked and it was extremely irritating’.

BS 2 ‘And for NHMRC, the chairs they appoint now, typically – well in many cases they don’t really know the science, that’s kind of how they appoint them’.

The process of appointing a strong technical or administrational chair seems to have been very well accepted by the respondents with significant past experiences of the NHMRC process.

##### Personal conflict

During the QUT research process, there was considerable personal conflict experienced by some panel members of the Basic Sciences group and there was consensus that personal conflict has a quite significant impact on the group dynamic as a whole and can directly influence the grading given to grants.

BS 5 ‘I mean it felt like it [conflict] affected it. Okay, so I think there were certainly a couple of personalities [in the QUT process] that were very strong on whether they liked a grant or not and I think when there was any – a strong debate about that between the strong personalities it was kind of – you could feel the group – well, I could feel the group siding with one or the other and you think ‘are you siding with a personality or the grant?’.

It is during this process that the role of the chair becomes vital in moderating this effect;

BS 6 ‘Yeah, I think in a regular GRP panel the chairperson would have been a bit more forthright in telling them to pull their head in – “bag it” - sort of got the message ‘don’t keep on flogging the horse’. Yeah there were some very strong people or people with very strong ideas’.

In direct contrast, the Public Health panel members all spoke highly of the group dynamic and the collegial atmosphere and clearly highlighted how this supportive environment enabled those less experienced or perhaps less confident members to present their views in an arena that made them feel safe to do so and in turn this presented the impression that the grants actually did receive a fair and considered peer review;

PH 8 ‘I think it was a very collegial panel. I certainly felt very supported. I felt confident to be able to express my views without feeling that someone was going to shout me down; though they did a couple of times, but that was alright. It was part of the process and it was really well done’.

##### Being the ‘expert’ in the room

Another aspect of group dynamic that was observed to be highly influential in relation to the discussion of a grant is the role that one person can take if they are seen by the group as being the expert in the room on a given topic;

BS 5 ‘Yeah, well, there was at least – there was one grant that I quite liked but [name]^4^ really did not and, as [pronoun] – I mean [pronoun] said multiple times “I’m an expert in this area” so I guess in those cases I didn’t really have a choice but to take [pronoun] opinion on board a bit more’.

This facet of the group dynamic was largely viewed by both panels as being a positive attribute of a panel discussion and something that is seen as enhancing the peer review of a grant. This point is made quite strikingly by one participant when discussing how this specific dynamic plays out in a review panel under usual circumstances and by highlighting how this then becomes rather dysfunctional due to the personal conflict in the room;

BS 3 ‘I suppose the other thing is that generally within our panel people recognise each other’s expertise and they respect other people’s expertise and people who don’t know anything about the area, don’t work in it, are not permitted to basically lay down the rules about what’s going to go on in a discipline they don’t work in, and that happened a lot in this committee’.

##### Panel size (QUT v NHMRC)

A key difference between the NHMRC process and the QUT process was panel size. The standard NHMRC panel formulation of 12 people was reduced for this research project to 7, and this was met with some concern by both panels. Specifically, although both agreed that the smaller panel size has some appeal and allowed for a more informal collegial discussion and the grants were discussed in a much speedier fashion;

PH 4 ‘you could actually go around the table without taking all day, which is just not possible on the full panel’.

Some interviewees felt that a smaller panel used in the QUT-simplified process allowed some of the more dominant personalities to take over the group and have an undue influence on the grant outcomes;

PH 3 ‘There’s some people who are really harsh about some things and some people who are very generous about some things but that’s just part of the nature and the larger the group size the more likely you are to have someone who was potentially influential, but the larger the group the less that will have an impact because it’s the dilution effect’.

Importantly, a number of members from both groups felt that the smaller panel size impacted on the breadth of expertise within the panel particularly when, as discussed previously, the experts left the room due to a conflict of interest;

PH 1 ‘In broader areas you’ve usually got enough expertise when you’ve got a panel of 10 or 12 people’.

Overall, the smaller panel size was poorly accepted with most respondents being more supportive of a larger panel;

BS 1 ‘I probably would have had a slightly bigger panel so you had more opportunity for debate’.

In summary, the QUT GRP process had a number of important variations to the standard NHMRC process. The panel size was smaller, and the chair of the Basic Sciences group was ‘technical’ rather than an expert in the field. There were no rebuttals, external reviews or secondary spokesperson. The grading of the grants for the QUT process took a three-tier-only approach, the conflict of interest rules were relaxed to a large extent, and no budget was provided. Although there was some agreement on certain principles across both panels, on the whole, the approaches taken to grant assessment were remarkably dissimilar.

### The role of research culture

This project allowed us to examine any major differences of the perspectives of the panellist in terms of their research culture. We use the term ‘research culture’ to loosely describe the ways in which researchers give priorities to particular aspects of research. While we are careful not to over extend our findings in this section, it was clear that there were overall traits that emerged in each panel’s dynamics.

For example, it was noticed that the Basic Sciences panel dynamics overall was at times combative and the language used tended to be blunt in particular the expressions of ‘pushing’ ‘killing’ and ‘sinking’ a grant. The panel expressed the value of a clear well-written grant but with the caveat that it be exclusive to one particular field which would then be expected to be reviewed only by experts from within that field. Emphasis was placed on the incremental value of basic science research with each proposal representing a small part of a ‘big picture’ and there was no need to have a proposal that could be immediately translational. The most striking aspects of a grant proposal for the Basic Sciences panel were novelty and innovation. The panel felt it was unnecessary to demonstrate value for money with more emphasis being placed on the likely contribution to the field and a strong sense of funding the person. With regard to the grading system, the panel overall felt that the QUT process was very similar to the NHMRC, with obviously outstanding proposals and clearly inadequate ones, both representing an easy decision, but both grading systems doing little to ease the decision-making for the grants finding themselves in the middle ground. Conflict of interest was highly contentious with a high degree of agreement that the regulation was absolutely essential and yet fundamentally flawed in terms of losing the required expertise from the room and still failing to adequately address the role of a personal agenda in promoting grant success.

The formulation of an experienced and appropriate panel remained a priority for all respondents particularly the matching of the required expertise to the very specific fields to which the grant would be applied and the panel size for this project was felt to be too small. In addition, the role of the spokesperson was seen as a significant responsibility given that the panel if unfamiliar with the field would follow the spokesperson. The role of track record for the Basic Sciences panel was considered to be vitally important, and the grant review was undertaken with a very ‘person centred approach’, judging as important criteria the contribution to the field that the applicant had made previously. Well-known large research teams were highly valued, and there was a strong bias to publications in specific journals that were considered to be the most eminent in the specific field. The group dynamic was reported at times to be combative, and most respondents admitted to the ability of a certain amount of ego and personal agenda to play a role in the eventual result for a grant application.

BS 1 ‘Yeah I think the biggest – you know, probably the tightest predictor of future success is your capacity to have done it before and part of that is what you’re looking for are people that can not only do the science but have the backbone and stamina to be able to write it up, get through the grant – the paper review process and get something published; that’s not a trivial thing’.

In contrast, the Public Health panel members commented on the high degree of collegiality within the panel and the respectfully supportive group dynamic. While there were some members recognised by others as being the clear experts, this was considered in a deferential way and not seen as something to be contested. This panel also deferred to the spokesperson for a decision on grants with which they were unfamiliar and this level of expertise was appreciated by the group. However, the spokesperson’s perspective on each grant appeared to be given full consideration rather than an automatic acceptance as absolute. This facet of the group dynamic was described as vitally important with regards to the configuration of a grant review panel which ideally would include one or two very experienced researchers in balance with more ‘early career researchers’ capable of providing a more contemporary or innovative perspective. Importantly, the distinction was made that the environment within the room needs to be one that allows a perhaps more inexperienced panel member to feel as though their contribution will be considered and valued. Both panels felt the QUT panel size was too small and the conflict of interest regulations were seen as problematic when the expert leaves the room; however, the PH panel tended to take a more relaxed approach to conflict of interest issues for the grant review process, with some degree of success.

In contrast to the Basic Science panel, for the Public Health panel, the lack of a budget was disconcerting and the members appeared to have a keen sense of ensuring a viable project with a tangible outcome be funded. The broader collegial nature of the Public Health panel also meant that the grant review process itself specifically addressed the grant’s potential contribution to the field and there was at no time acknowledgement of the person being funded rather than the grant. When track record did come under consideration, it was in a more comprehensive way, and although publications were acknowledged as important, equally so was the person’s overall career achievements. This grant review panel very clearly articulated assessing the quality of the grant first and foremost, and although track record was their second consideration, it was not necessarily dependent on a citation index. There was certainly some acknowledgement of the disadvantage that can be experienced by early career researchers when the playing field is not level which was expressed quite frankly in the Basic Sciences panel.

PH 6 ‘So a lot of that Canberra process I think is people - you know, they feel it’s an honour to serve on this group, and so they have to justify their existence and also show to their peers how wise and clever and smart they are. So a lot of the comments and feedback - and they are not actually about the grant, they are about the individual giving them – “look how widely read I am. You know, I am attacking this grant because I am so clever”, and I think that’s an issue. Whereas our group was just, “Come on, let’s get to the bottom of this. Is there any good or not?” We were a lot less formal and our agenda was a healthier one’.

## Discussion

The research reported here was based on a qualitative approach using in-depth interviews with a relatively small number of respondents. While this approach was entirely suited to the methodology required for research of this kind, the limitations are well recognised. For example, we would not claim that the results of this research can be generalised to all participants taking part in grant review processes. We do believe, however, that the consensus between many of our respondents on a number of points we have discussed gives us comfort to believe that our findings capture beliefs, opinions and decision-making processes that are shared beyond this particular project.

We believe that our research provides a valuable ‘lens’ into an area that has previously been neglected. While the field of commentary on the peer review process is vast (see reviews by Lee et al. [13]; van Arensbergen et al. [14] and Guthrie et al. [15]), other examinations have mostly relied on quantitative methods of data collection and analysis. The research reported here provides a qualitative understanding of the peer review process, and it adds to the limited literature in this field, especially that of Mow [9, 16, 17], on examining the ‘black box’ representing the judgements, values and decision-making of members of grant review panels. It also complements the work of Abdoul et al. [18]. However, there are two standout features that separate the present study from that of others. Firstly, we had the opportunity in this project to compare and contrast the considerations of two distinctly different ‘cultures’ in health and medical research: Basic Sciences and Public Health. Secondly, our use of a simplified reviewing process allowed many of the panel members to comment on its usefulness compared to the more conventional systems, which in Australia, are usually the domain of the Australian Research Council (ARC) or the National Health and Medical Research Council (NHMRC).

Despite the different backgrounds, there were many areas of agreement between members of the two cultures when judging the quality of grant proposals. Foremost of these was the proposal’s clarity and significance. The ability of a proposal to ‘speak to’ the reader and effectively convey good science was considered uppermost by most of the panel members. This quality may result from the sheer volume of proposals reviewers have negotiate as part of their GRP membership. For NHMRC panels, this is often over 100 proposals, and for ARC even more. Proposals that are poorly written are likely to alienate reviewers.

Another feature that participants commonly discussed was the track record of the applicants. This was judged on number and quality of publications (in journals with high impact factors). The emphasis on these attributes raises questions about the extent to which early career researchers in the application process may be disadvantage by virtue of them not being able to have published in quantities likely to impress panels, or to have existing papers cited by others. In other words, a preoccupation with current interpretations of track record in the GRP process is likely to favour senior researchers. While it is true that fellowship funding is available for early career researchers outside of main NHMRC and ARC project schemes [19], these latter sources of funding are considered to be the most prestigious. One area in which the research cultures showed most difference concerned the interpretation of ‘good science’. For the Basic Science panel members, this was defined mainly as qualities leading to breakthroughs and paradigm-shifts. On the other hand, for Public Health panellists, high quality was often represented by translation and incremental development of ideas (so-called scaling up) from small to larger populations with ‘tangible outcomes’.

The simplified reviewing process in which the panellists took part omitted a number of features often included in conventional processes. This provided an opportunity for respondents to comment on whether this compromised the rigour of the simplified process. For example, the lack of a rebuttal allowed respondents to consider the merits of the rebuttal. Both groups believed that the rebuttal was unlikely to influence the decision of the primary spokesperson. And, given that the views of the primary spokesperson were considered to be crucial in the success of a proposal, the rebuttal—as an opportunity to further develop the proposal—may be redundant. However, where external assessments are obtained, the opportunity for applicant response or rebuttal is required under Administrative Law provisions to ensure procedural fairness. Another key omission in the simplified review process was budget. While budget was not considered a major issue for the Basic Science panellists, the Public Health panellists saw it to be crucial, especially in terms of value for money. This may reflect a greater belief in fields of public health that health interventions and services need to be not just effective but also cost effective. The increasing appearance of a health economics component on many public health grant proposals is further evidence of this.

One of the most striking outcomes of this research was the discussion by both groups of the problems with proposals getting a ‘fair review’, and with that, the central role played by the primary spokesperson. In the views of many participants, the peer review process of the GRP was hampered because grants were not always reviewed by ‘peers’. This results from GRP members often having conflicts of interest with applications and therefore not able to be part of the appraisal process. Many members spoke of panel experiences where primary spokespeople were clearly not expert in the field of the proposal. But without the confidence of expertise, a primary spokesperson is unlikely to award an application a high score, thereby ‘sinking’ or ‘killing’ it. Members talked about how they and others elevated their score to compensate for this possibility. This and other ways of ‘gaming the score’ were considered inevitable.

One of the aims of this study was to compare a simplified review process with more conventional processes. In general, participants were supportive of the simplified process. In particular, they liked the more nuanced definition of ‘conflict’ adopted by the simplified review—allowing conflicts to be rated high, medium and low and with this, various degrees of distance from the review process (for example, participants with low conflict were granted an opinion on the proposal after the panel had reached a rating, which may result in an up or down revision). Another feature of the simplified review process was awarding proposals a grade of gold (should fund), silver (could fund) and bronze (should not fund) instead of the NHMRC 1 to 7 rating. The extent to which the simplified process removed the grey middle ground was, however, a point of discussion. One suggestion was expanding from three categories to four by including a silver A or silver B grading.

Our comparison of research cultures raises a number of important issues. Cultural homogeneity within a GRP is likely to create the same criteria of the judgement of so-called excellence [20]. However, in reality, some panels are likely to comprise researchers from a variety of backgrounds: for example, basic sciences, clinical practice and population health. The application of different criteria of what is ‘excellence’ potentially leads to difficulties in agreement of rating and ranking, no doubt giving rise to the idea that GRP processes are a lottery. More complication is added when grants are considered using different assumptions about research process. An example here would be research involving Australian Indigenous communities, where community consultation and participation is part of generating hypotheses (as in action research).

## Conclusion

In her book *How Professors Think*, Lamont [21] states ‘Peer review is secretive. Only those present in the deliberative chambers know what happens there.’ In this paper, we have attempted to throw light on the so-called black box of peer review and to reveal some of the associated secrecy and introduce transparency [22]. We believe that the more light shone on the ‘black box’, the greater the likelihood of transparency of process and the better will be the appreciation of outcome. In drawing some conclusions from our work, we make the following points. Firstly, despite the provision of clear guidelines for peer review—including full descriptions of the criteria by which proposals should be rated—reviewing processes are likely to be subjective to the extent that different reviewers apply different rules. The dynamics of GRP panels provide an opportunity for the differences in opinion and judgement to be played out. But as our research suggests, academic engagement in GRPs is accompanied by a variety of dramatics and theatrics that are difficult to remove in a human environment. Secondly, as funding for research becomes even more competitive, peer review processes are likely to become more contested simply because there will be more at stake. Inevitably, the process of peer review will come under much more scrutiny. Thirdly, the points raised, so far, have given justification for further research on the peer review process, especially of a kind that taps more deeply into the ‘black box’ of peer review.

## Abbreviations

ARC: Australian Research Council
GRP: Grant review panel
NHMRC: National Health and Medical Research Council

## Footnotes

1 BS 6 refers to Basic Sciences panel, respondent 6; similar rules apply to PH notation

2 The notation of an ellipse […] indicates that the quote has had some section removed for easier reading

3 [pronoun] indicates that gender pronouns have been deleted

4 [name] indicates that a participant name has been deleted
